# The Evolving Role of Extracorporeal Carbon Dioxide Removal in Acute Respiratory Failure: A Narrative Review

**DOI:** 10.7759/cureus.105631

**Published:** 2026-03-21

**Authors:** Safiya Sherrin, Jasmine Kaur, Vaibhav Taneja, Wafabi Mustafa, Ketan Garg, Kumar Kunal

**Affiliations:** 1 Critical Care Medicine, Dayanand Medical College and Hospital, Ludhiana, IND; 2 Critical Care Medicine, MY Hospital, Mohali, IND; 3 Anaesthesiology, Goa Medical College, Goa, IND; 4 Pathology, Jyoti Gupta Clinic, New Delhi, IND; 5 Anaesthesia and Critical Care, Military Hospital, Jaipur, IND

**Keywords:** cannula, hemolysis, hypercapnia, pulmonary disease, respiratory distress syndrome, thrombosis

## Abstract

Extracorporeal carbon dioxide removal (ECCO₂R) has emerged as a supportive therapy for patients with acute respiratory failure, particularly when refractory hypercapnia and respiratory acidosis persist despite standard management. This narrative review summarizes the physiological rationale, current evidence, and clinical applications of ECCO₂R in modern critical care practice. ECCO₂R removes carbon dioxide directly from the bloodstream, thereby facilitating lung-protective ventilation strategies and potentially reducing ventilator-induced lung injury. The technique has been primarily evaluated in patients with acute respiratory distress syndrome (ARDS) and acute exacerbations of chronic obstructive pulmonary disease (AECOPD). In these settings, ECCO₂R may enable the use of lower tidal volumes and reduced airway pressures, and in selected patients, it may decrease the need for invasive mechanical ventilation. Although ECCO₂R has been shown to improve carbon dioxide clearance, correct respiratory acidosis, and optimize ventilatory parameters, randomized trials have not demonstrated a definitive mortality benefit. Its clinical application is also limited by device-related complications, including bleeding, thrombosis, and hemolysis. Furthermore, outcomes are influenced by technical factors such as cannula configuration, blood flow rate, membrane efficiency, and anticoagulation strategy. Ongoing technological advancements and well-designed clinical trials are needed to better define the role of ECCO₂R in contemporary critical care practice.

## Introduction and background

Clinical context

Acute respiratory failure remains a cornerstone challenge in critical care medicine. Patients presenting with Acute Respiratory Distress Syndrome (ARDS) or Acute Exacerbation of Chronic Obstructive Pulmonary Disease (AECOPD) often face a precarious balance: the need for mechanical ventilation to support gas exchange versus the risk of ventilator-induced lung injury (VILI) caused by high pressures and volumes. While conventional invasive mechanical ventilation (IMV) is the standard of care, it is limited by its potential to perpetuate secondary lung inflammation. Consequently, there is a growing clinical focus on "ultra-protective" ventilation strategies, which aim to reduce tidal volumes and respiratory rates to the minimum necessary for patient safety [1].

Extracorporeal Carbon Dioxide Removal (ECCO₂R) has emerged as a targeted therapy to facilitate these ultra-protective strategies. It is vital to distinguish ECCO₂R from Extracorporeal Membrane Oxygenation (ECMO). ECMO is designed for comprehensive support and requires high blood flow (often >3 L/min) to provide both oxygenation and CO₂ removal. In contrast, ECCO₂R is a low-flow extracorporeal technique (typically 400-600 mL/min) specifically engineered to decarboxylate blood. By offloading the primary burden of CO₂ removal to an external artificial lung, ECCO₂R allows the native lungs to rest, effectively managing hypercapnic respiratory failure and the associated respiratory acidosis [2].

**Figure 1:**
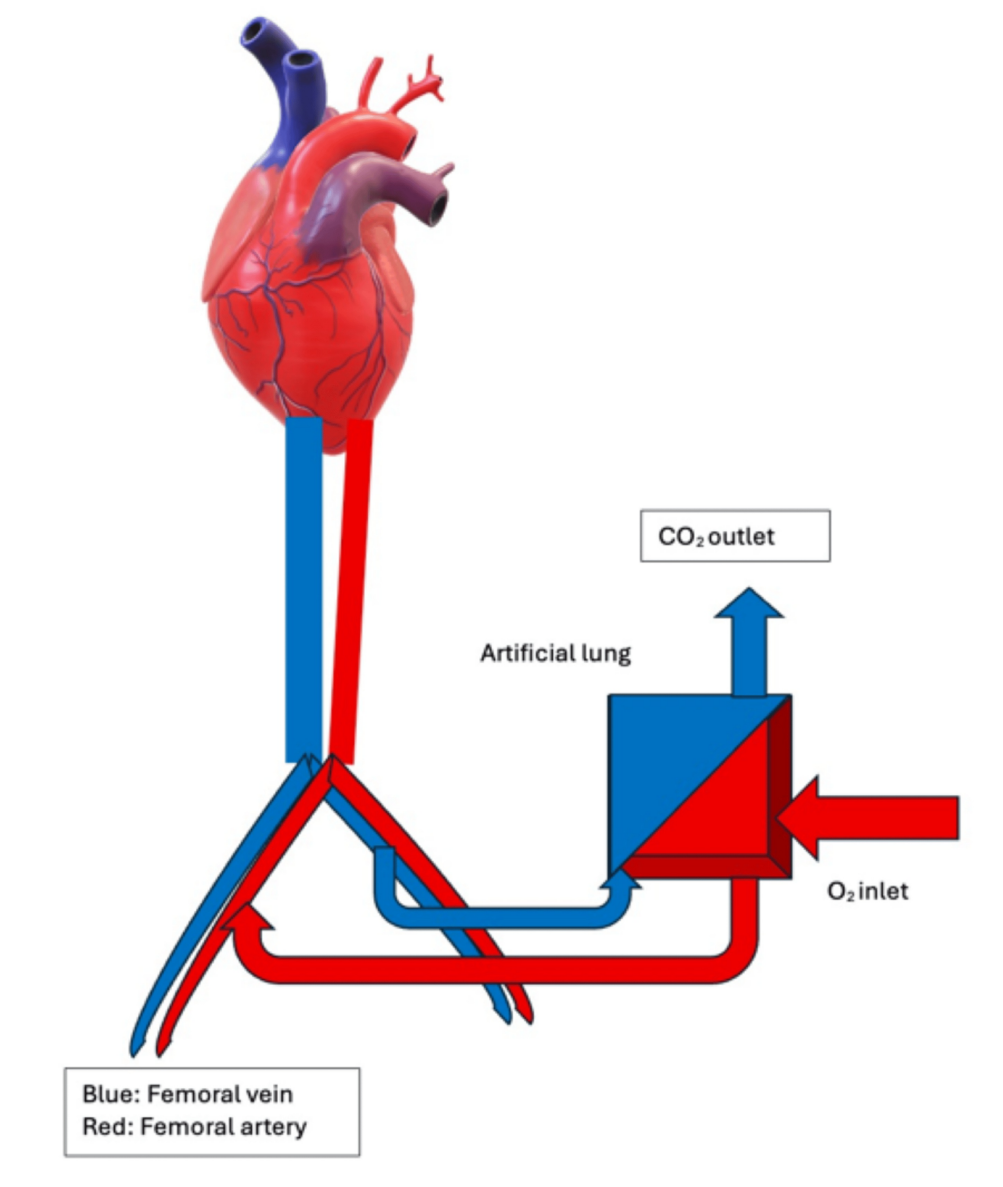
Arterio-venous extracorporeal carbon dioxide removal (ECCO₂R) system. The image demonstrates an arterio-venous extracorporeal carbon dioxide removal (ECCO₂R) system using femoral cannulation. Deoxygenated blood is withdrawn from the femoral vein (shown in blue) and directed through an extracorporeal circuit to a membrane lung (artificial lung). Within the membrane, carbon dioxide is removed via a sweep gas system, with oxygen entering through the inlet and carbon dioxide exiting through the outlet. The partially purified blood is then returned to the systemic circulation via the femoral artery (shown in red). This pumpless system utilizes the patient’s native arterial pressure gradient to drive blood flow, thereby facilitating CO₂ removal and reducing hypercapnia. The figure is prepared using Microsoft Word by combining simple shapes and inserted illustrative elements, including a 3D heart model. No AI-generated tools were used in its creation.

Extracorporeal carbon dioxide removal (ECCO₂R) was first introduced in 1977 as a technique aimed at controlling arterial carbon dioxide levels while allowing reductions in mechanical ventilatory support [1,2]. The primary objective of this approach was to facilitate lung rest by reducing ventilator settings, particularly in patients with acute respiratory failure. It allows the circulation of blood from the internal jugular vein in a venovenous configuration, wherein blood is withdrawn from the internal jugular vein and passed through a membrane. The system facilitates sweep gas flow, allowing removal of CO₂. A pump then returns the blood back into the internal jugular vein. Another configuration is arteriovenous femoral cannulation, in which blood is withdrawn from the femoral vein and passed through a membrane that removes CO₂, functioning as an artificial lung. The blood is then returned to the femoral artery, thereby reducing the partial pressure of CO₂ in the blood, as shown in the graphical representation (Figure 1) [1-5].

Physiological basis

The efficacy of ECCO₂R relies on the high CO₂ content of venous blood (approximately 45-50 mL/100 mL). By diverting a small fraction of cardiac output through a membrane lung, clinicians can remove 150-200 mL/min of CO₂, the typical metabolic production rate, without the need for the high pump speeds associated with ECMO [3].

Early clinical applications of ECCO₂R were largely exploratory, aimed at proving that extracorporeal decarboxylation could successfully manage respiratory acidosis in patients who were failing conventional non-invasive ventilation (NIV). It was demonstrated that by utilizing an extracorporeal circuit, clinicians could successfully avoid endotracheal intubation in a subset of respiratory failure patients, thereby reducing the complications associated with invasive mechanical support [2,4,6]. 

Respiratory failure is broadly categorized based on gas exchange deficits. Hypercapnic (Type II) respiratory failure is a failure of the "bellows" function of the respiratory system. In conditions such as AECOPD, the lungs are unable to clear CO₂, resulting in hypercapnia and a drop in pH (acidosis). Because ECCO₂R uses a membrane lung to strip CO₂ directly from the blood, it addresses the root cause of the acidosis without requiring the patient to increase their minute ventilation [7].

Conversely, hypoxemic (Type I) respiratory failure involves the lung parenchyma itself. In ARDS, the alveolar-capillary membrane is damaged, preventing adequate oxygen transfer. While ECCO₂R does not directly improve oxygenation, it serves a protective role: by removing CO₂ extracorporeally, the ventilator settings can be significantly "de-escalated." This reduction in ventilator-induced lung injury (VILI) is the primary clinical objective when using ECCO₂R in hypoxemic patients [7].

Current status and objectives

Despite its potential, the optimal role of ECCO₂R in modern intensive care remains a subject of ongoing investigation. While early applications focused on severe hypoxemic and hypercapnic failure, current research is evaluating its efficacy in preventing intubation for patients failing non-invasive support [8].

This narrative review aims to synthesize the physiological basis, technological advancements, and clinical outcomes surrounding ECCO₂R to provide a framework for its application in contemporary practice.

## Review

Literature search strategy

A narrative literature review was conducted to summarize the current evidence regarding extracorporeal carbon dioxide removal (ECCO₂R). The literature search was independently performed by two investigators (first and second authors) using multiple electronic databases, including PubMed, Elsevier, Google Scholar, and Web of Science. The search strategy used keywords such as ECCO₂R and CO₂ removal. Articles published in English over the last 15 years and available as freely accessible full texts were screened. Randomized controlled trials, observational studies, meta-analyses, and relevant review articles addressing the clinical applications, outcomes, and physiological principles of ECCO₂R were included based on relevance to the topic. As this study was designed as a narrative review, a formal systematic review framework including PRISMA-based study selection and risk-of-bias assessment was not undertaken.

Evolution

ECCO₂R has been explored as a supportive therapy in ARDS and acute hypercapnic respiratory failure, particularly during AECOPD. Early ECCO₂R systems used a pumpless arterio-venous (AV) configuration that diverted a portion of the patient’s cardiac output through a femoral AV circuit. Clinical studies demonstrated a significant reduction in arterial CO₂ levels and improvement in respiratory acidosis [3,4]. However, complication rates were substantial, including bleeding (18-47%), thrombosis (0-20%), and limb ischemia (4.5-22%), which limited widespread clinical adoption [5,6].

Subsequently, pumped venovenous (VV) ECCO₂R systems were developed, typically using double-lumen cannulas inserted in the femoral or jugular veins. Experimental and early clinical studies showed that this technique could effectively control hypercapnia and respiratory acidosis in patients with ARDS and AECOPD [7,8]. More recently, ECCO₂R systems have been integrated with renal replacement therapy platforms, facilitating their use in intensive care settings. Observational reports suggest that this approach may reduce intubation rates in selected AECOPD patients and improve respiratory parameters when used alongside non-invasive ventilation [9-11].

Despite these physiological improvements, clinical outcomes remain variable. A meta-analysis including four randomized controlled trials and five observational studies involving 1,173 patients with acute respiratory failure (ARF) due to COPD or ARDS reported no mortality advantage with ECCO₂R. Although reductions in ICU stay and improvements in gas exchange and pH were observed, hospital length of stay was increased [12]. Additional smaller studies and case reports have also demonstrated the physiological effectiveness of ECCO₂R. For example, a pediatric case report described successful correction of severe hypercapnia and respiratory acidosis, preventing the need for invasive mechanical ventilation and serving as a bridge to lung transplantation [13]. Similarly, a study involving 159 patients with acute respiratory failure reported significant improvement in hypercapnic acidosis and reduced ventilatory requirements within four hours of ECCO₂R initiation, highlighting its potential role in rapid CO₂ clearance even though long-term survival benefits remain uncertain [14].

Indications and response

ARDS

In patients with ARDS, ECCO₂R has primarily been investigated as a strategy to facilitate lung-protective or ultra-protective ventilation. Efforts to reduce tidal volume, plateau pressure, and driving pressure are often limited by the development of severe respiratory acidosis. By directly removing carbon dioxide from the bloodstream, ECCO₂R may enable further reductions in ventilator settings while maintaining adequate gas exchange [15,16].

The SUPERNOVA study demonstrated that ECCO₂R can be safely used to achieve ultra-low tidal volume ventilation (3-4 mL/kg) while maintaining effective control of CO₂ levels [15]. Similarly, the REST trial compared standard ventilation (6-8 mL/kg) with ultra-low tidal volume ventilation supported by ECCO₂R. Although tidal volume separation between groups was achieved and comparable gas exchange was maintained, the study did not demonstrate a significant difference in 90-day mortality. Moreover, patients in the ECCO₂R group experienced higher rates of complications, including bleeding and intracranial hemorrhage, which led to early termination of the trial and reduced the potential advantage of lung-protective ventilation [16].

Although large randomized trials have reported neutral results, several observational studies and case reports suggest potential physiological and clinical benefits of ECCO₂R in selected ARDS patients. For example, a study including 75 ARDS patients treated with ECCO₂R reported improvements in survival rates and reductions in the duration of mechanical ventilation and hospital stay [17]. Another study involving 28 ARDS patients and 20 patients with status asthmaticus demonstrated effective CO₂ removal using ECCO₂R with blood flow rates of 200-450 mL/minute [18]. Individual case reports have further illustrated the potential role of ECCO₂R as a bridging strategy. In one report involving a 44-year-old COVID-19 patient with severe hypercapnia, ECCO₂R significantly improved PaCO₂ levels and corrected acidosis prior to transfer to an ECMO center, where the patient ultimately survived after a prolonged hospital stay [19]. Collectively, these findings suggest that ECCO₂R may provide meaningful physiological benefits in selected patients with severe respiratory failure [5,19].

AECOPD

In patients with AECOPD, non-invasive ventilation remains the standard of care. ECCO₂R has been evaluated as an adjunctive therapy for patients who fail to tolerate or respond adequately to NIV. It has been suggested that ECCO₂R can effectively correct respiratory acidosis and reduce the work of breathing. However, improvements in ventilator-free days or mortality have not been consistently demonstrated, and higher mortality has been reported despite combining ECCO₂R with NIV [20].

In one study involving 25 COPD patients, venovenous ECCO₂R avoided the need for invasive mechanical ventilation in approximately 50% of patients. Nevertheless, device-related complications occurred in nearly one-third of the cohort [21]. Furthermore, ECCO₂R has also been explored as a bridge to lung transplantation, enabling selected patients to remain awake and ambulatory before surgery [22].

Although clinical outcomes remain heterogeneous, technical factors appear to influence treatment efficacy. Available data suggest that higher-flow systems (500-1000 mL/minute) achieve more efficient CO₂ clearance compared with lower-flow devices (<500 mL/minute) [5,23].

Key randomized trials and major observational studies evaluating ECCO₂R in acute respiratory failure are summarized in Table 1.

**Table 1:** Summary of major clinical studies evaluating extracorporeal carbon dioxide removal (ECCO₂R) in patients with acute respiratory failure. ECCO₂R: Extracorporeal Carbon Dioxide Removal; ARDS: Acute Respiratory Distress Syndrome; COPD: Chronic Obstructive Pulmonary Disease; AECOPD: Acute Exacerbation of Chronic Obstructive Pulmonary Disease; NIV: Non-Invasive Ventilation; ICU: Intensive Care Unit; PaCO₂: Partial Pressure of Carbon Dioxide in Arterial Blood; PaO₂: Partial Pressure of Oxygen in Arterial Blood; PaO₂/FiO₂: Ratio of Arterial Oxygen Partial Pressure to Fraction of Inspired Oxygen; FiO₂: Fraction of Inspired Oxygen; RCT: Randomized Controlled Trial.

Study	Design	Population	Key Findings
Tiruvoipati et al. [14]	Multicenter, multinational retrospective cohort study	Patients with acute respiratory failure treated with Hemolung ECCO₂R	Significant improvement in hypercapnic acidosis with reduction in PaCO₂ and improvement in pH within hours of ECCO₂R initiation. Survival to ICU discharge was 41%, highest in status asthmaticus (86%), followed by ARDS (52%) and COVID-19 ARDS (31%). Younger age (<65 years), non-COVID ARDS, and higher PaO₂/FiO₂ at initiation were associated with better survival.
SUPERNOVA Trial [15]	Multicenter feasibility study	Moderate–severe ARDS	ECCO₂R enabled ultra-low tidal volume ventilation (3–4 mL/kg) while maintaining adequate CO₂ control.
REST Trial [16]	Randomized controlled trial	ARDS patients	No significant difference in 90-day mortality; higher rates of bleeding and intracranial hemorrhage observed with ECCO₂R.
Inal et al. [17]	Retrospective case-control study	Severe hypercapnic respiratory failure due to COPD or ARDS	ECCO₂R improved survival (68% vs 58%), reduced duration of invasive mechanical ventilation, and shortened ICU stay compared with conventional therapy.
Braune et al. (ECLAIR study) [21]	Multicentre case-control study	AECOPD with hypercapnic respiratory failure refractory to NIV	ECCO₂R avoided invasive mechanical ventilation in 56% of patients, though device-related complications occurred in 44%, mainly major bleeding. No difference in ICU stay or 90-day mortality was observed.
Zhang et al. [18]	Multicenter retrospective descriptive study	ARDS and status asthmaticus patients treated with low-flow ECCO₂R	Low-flow ECCO₂R (200–450 mL/min) resulted in a significant reduction in PaCO₂ within 24 hours (~25% decrease) with clinically acceptable outcomes; mortality differed between ARDS and asthma groups.
Zhou et al. [12]	Systematic review and meta-analysis (4 RCTs + 5 observational studies; n = 1173)	Acute respiratory failure due to COPD or ARDS	No significant mortality benefit with ECCO₂R; however, lower intubation and tracheotomy rates, shorter mechanical ventilation duration, and improved physiological parameters (pH, PaCO₂, PaO₂, respiratory rate) were observed, particularly in COPD patients.

Factors affecting efficacy

Cannula

The choice and placement of the cannula are factors determining ECCO₂R performance. Since ECCO₂R functions at lower blood flows compared to ECMO, smaller cannulas (13-14 Fr) are used that decrease invasiveness and vascular injury [24]. However, smaller diameters restrict the achievable blood flow, and shear stress may increase when higher flow rates are attempted. Elevated shear stress can cause hemolysis and affect effective CO_2_ removal [5]. For achieving effective CO_2_ clearance, it is crucial to maintain adequate blood flow, generally about 400-450 mL/minute. To increase blood flow and decrease recirculation, two single-lumen catheters instead of one double-lumen device are used in some systems. These are commonly placed in the jugular and femoral veins. Efficiency is significantly reduced by recirculation, because treated blood is drawn back to the circuit instead of systemic circulation. The extent of recirculation is affected by the catheter type, length, position, and duration of use. Recirculation is minimized by placement in the internal jugular or subclavian vein with the tip near the right atrium. Femoral placement results in higher rates of recirculation and comparatively reduced efficiency [5].

Pumps

Pump design significantly affects blood flow stability and hemocompatibility. Several ECCO₂R systems adapt roller pumps from renal replacement therapy (RRT) or centrifugal pumps from ECMO circuits. Roller pumps are usually restricted to flows below 500 mL/minute, limiting the capacity of CO_2_ removal. On the contrary, centrifugal pumps designed for high-flow ECMO may result in excessive shear stress when low ECCO₂R flow rates are used. This increases the risk of haemolysis [5,25]. New low-flow pump technologies are being developed to increase performance. Some integrated pump-membrane systems incorporating rotating impellers can function effectively at flow rates below 250 mL/minute while maintaining adequate hemolysis levels. These designs are intended to enhance CO_2_ extraction while decreasing mechanical blood trauma [5,26,27].

Membranes

Membrane performance depends on the diffusion gradient, membrane surface area, contact time between blood and membrane, and structural design. CO_2_ removal increases with higher sweep gas flow and greater blood CO_2_ content. Modern membrane lungs range from approximately 0.8 to 3 m². Smaller membranes reduce priming volume and anticoagulation requirements but may increase resistance and pressure drop. Membrane configuration also affects efficiency. Parallel-plated membranes tend to achieve higher CO_2_ removal at moderate to high sweep gas flows, whereas circular fibre arrangements may perform better at very low gas flows, though this is less relevant clinically. For meaningful comparison between devices, CO_2_ removal (VCO_2_) should be standardised relative to membrane surface area (mL/min/m²) [5,28].

Materials

Early ECCO₂R membranes were constructed from microporous polypropylene fibres. These membranes enabled efficient gas exchange through microscopic blood-gas interfaces but were prone to plasma leakage over time. To overcome this limitation, non-microporous poly-4-methyl-1-pentene (PMP) membranes were introduced. PMP fibres offer improved durability, superior gas exchange capacity, and enhanced biocompatibility with significantly reduced plasma leak risk. Gas transfer efficiency has been further optimised by arranging fibres into complex mat configurations and directing blood flow along the outside of the fibres. This perpendicular flow pattern shortens the diffusion pathway and enhances mass transfer compared with parallel flow designs [5].

New surfaces

Artificial surfaces within extracorporeal circuits activate inflammatory and coagulation pathways, potentially compromising device performance. To address this, modern ECCO₂R systems incorporate bioactive surface coatings. Heparin-based coatings and nitric oxide (NO)-releasing materials mimic endothelial function and reduce platelet activation and clot formation. Some systems deliver NO directly into the membrane circuit to further inhibit thrombosis [29]. Emerging strategies include endothelialization of artificial surfaces to replicate the natural vascular lining, potentially reducing reliance on systemic anticoagulation. Hollow fibre membranes coated with carbonic anhydrase (CA) enhance the conversion of bicarbonate into CO_2_, increasing the diffusion gradient at the membrane surface and improving CO_2_ removal efficiency [30]. These CA-coated membranes also improve haemocompatibility by markedly reducing platelet adhesion. Experimental approaches combining CA-coated membranes with acidic sweep gases have demonstrated further enhancement of CO_2_ clearance. Intravascular gas exchange devices represent a promising future direction [31].

Measuring device VCO_2_ to assess membrane performance

Accurate assessment of device performance requires reliable measurement of CO_2_ removal (VCO_2_) [32]. This can be achieved by calculating transmembrane CO_2_ content differences in blood or by measuring CO_2_ levels in the effluent gas using infrared sensors. For meaningful comparison between devices, VCO_2_ should be standardised to inlet PCO_2_ and blood flow conditions. Reporting performance relative to membrane surface area (e.g., mL/min/m²) allows more consistent benchmarking across systems [33].

Combined CO_2_ removal (lung dialysis) with renal support

Since most blood CO_2_ is transported as bicarbonate, strategies targeting bicarbonate handling can enhance CO_2_ removal. ECCO₂R may be combined with continuous renal replacement therapy (CRRT), integrating a gas exchange membrane into the renal circuit. This approach can reduce PaCO_2_, improve pH, and stabilise haemodynamics in patients with respiratory and renal failure. Membrane placement within the circuit and dialysate composition influence removal efficiency, although optimal configurations remain under investigation [34,35].

Contraindications

Although ECCO₂R has shown potential physiological benefits in selected patients with acute respiratory failure, careful patient selection is essential. Several clinical situations may limit or preclude the use of ECCO₂R. Patients who meet indications for extracorporeal membrane oxygenation (ECMO), such as those with severe or refractory ARDS or significant right ventricular failure, may not be suitable candidates for ECCO₂R, as ECMO may provide more comprehensive cardiopulmonary support. Additionally, the requirement for systemic anticoagulation during ECCO₂R therapy makes the procedure unsuitable in patients with contraindications to anticoagulation, including those at high risk of bleeding [36]. Other factors that may limit the use of ECCO₂R include difficulties with vascular access and the presence of severe comorbidities associated with poor long-term prognosis. Patients with an expected survival of less than one year or those with advanced end-stage disease may derive limited benefit from ECCO₂R therapy. Furthermore, patient preference should be considered, particularly in cases where individuals decline invasive respiratory support or extracorporeal therapies [36]. In patients with AECOPD, additional exclusion considerations include severe functional limitation, cachexia, poor quality of life, or situations where the patient is not considered a candidate for mechanical ventilation. These factors should be carefully evaluated when determining the appropriateness of ECCO₂R therapy [36]. 

Expert consensus guidelines on ECCO₂R

The expert consensus report on ECCO₂R by a European round table meeting involving 14 experienced intensivists aimed at clarifying how ECCO₂R is currently applied in clinical practice, particularly in patients with acute respiratory distress syndrome (ARDS) and acute exacerbations of chronic obstructive pulmonary disease (AECOPD), and to identify areas of agreement regarding indications, treatment targets, and weaning strategies [36].

Using a modified Delphi method, experts participated in pre-meeting and post-meeting surveys alongside structured discussions. ARDS was considered the primary indication for ECCO₂R by most participants, while ae-COPD was the second main indication. In ARDS, the principal objective of ECCO₂R was to enable ultra-protective lung ventilation (UPLV) by controlling hypercapnia and respiratory acidosis [15]. The group reached consensus that elevated driving pressure (≥14 cmH_2_O) and plateau pressure (≥25 cmH2O) were key criteria for initiating ECCO₂R [37]. Other important parameters included low pH (<7.25), elevated PaCO_2_, and high respiratory rate. Treatment targets during ECCO₂R included reducing driving pressure below 14 cmH_2_O, maintaining plateau pressure below 25 cmH_2_O, keeping respiratory rate under 20-25 breaths per minute, and achieving pH above 7.30 [38].

In AECOPD, ECCO₂R was mainly recommended for patients at risk of non-invasive ventilation (NIV) failure or to facilitate early extubation after intubation. Failure of PaCO_2_ and respiratory rate to improve during NIV was considered a strong indication for ECCO₂R initiation. Treatment goals focused on patient comfort, pH improvement (generally >7.30-7.35), reduction in respiratory rate, partial reduction of PaCO_2_ (10-20%), and successful weaning from NIV or mechanical ventilation [36].

Consensus was also achieved on weaning protocols for both ARDS and AECOPD, emphasizing clinical stability and gas exchange improvement before discontinuation. Intravenous unfractionated heparin was identified as the preferred anticoagulation strategy. Overall, the panel concluded that ECCO₂R may serve as a valuable supportive therapy in selected ICU patients, but emphasized the need for further high-quality randomized clinical trials to strengthen the evidence base [36].

The 2022 European roundtable meeting on ECCO₂R updated recommendations on the clinical application of ECCO₂R in acute respiratory failure. The report was developed by an international panel of experts using a structured consensus methodology to address patient selection, technical management, monitoring, and safety considerations [39].

The panel emphasised that ECCO₂R is primarily intended to facilitate lung-protective or ultra-protective ventilation in patients with moderate-to-severe ARDS who develop refractory hypercapnia despite optimised ventilator settings. By enabling reductions in tidal volume, plateau pressure, and driving pressure, ECCO₂R may help minimize ventilator-induced lung injury. However, the document stresses that current evidence does not yet demonstrate a clear survival benefit, and its use should be restricted to experienced centres [39].

In patients with AECOPD, ECCO₂R may be considered when non-invasive ventilation fails or to support early extubation. Careful monitoring of gas exchange, haemodynamics, and anticoagulation is essential, as bleeding and thrombosis remain significant complications. The consensus highlighted the importance of standardised reporting of CO_2 _removal rates, blood flow, and device performance to enable meaningful comparison between systems. Ultimately, the report concluded that while ECCO₂R is a promising adjunctive therapy, further high-quality randomised trials are required to define its precise role in routine clinical practice [39].

The expert consensus guidelines from the European Society of Intensive Care Medicine address extracorporeal life support (ECLS), including ECCO₂R, as part of non-pharmacological respiratory support strategies for ARDS. The panel assessed whether ECCO₂R enables ultra-protective ventilation by facilitating further reductions in tidal volume and mechanical power while maintaining acceptable gas exchange. Although ECCO₂R can effectively remove carbon dioxide and theoretically reduce ventilator-induced lung injury, current evidence does not demonstrate a clear mortality benefit in unselected ARDS populations. Concerns remain regarding complications such as bleeding, thrombosis, device-related adverse events, and resource intensity [40].

The panel emphasizes that ECCO₂R should not be routinely applied outside experienced centers and clinical trials. Patient selection is critical, particularly in moderate ARDS, where hypercapnia limits lung-protective ventilation. The certainty of evidence was graded as low to moderate due to heterogeneity of studies and limited high-quality randomized trials. As a result, the guideline does not issue a strong recommendation for routine ECCO₂R use but highlights it as a potential adjunct in carefully selected cases. Future research priorities include defining optimal patient phenotypes, timing of initiation, standardized ventilator protocols, and evaluation of long-term patient-centered outcomes [40].

Future advances and future research

The future of ECCO₂R is uncertain, mainly due to mixed clinical outcomes and significant complications related to current devices. ECCO₂R demonstrated promising results as supportive therapy in patients with acute respiratory failure. But, its wide implementation depends on improvement in safety, refining technical performance, and evidence of patient-centered advantages. Future priorities are decreasing hemolysis, thrombosis, and bleeding, along with optimizing blood flow and sweep gas settings to increase efficiency. Standardizing monitoring strategies and reliable methods should be developed for comparing device performance [5,41].

Hemolysis is a serious complication of extracorporeal support; it showed an independent association with higher mortality rates. The principal driver of hemolysis is shear stress, which is generated when blood circulates through artificial circuits. Shear stress results from continuous flow patterns and contact with artificial surfaces. Hemolysis rates are affected by many factors, which include catheter size and design, rate of blood flow, pump type (roller versus centrifugal), membrane properties, anticoagulation strategy, and overall configuration of ECCO₂R [5,42,43].

The most reliable marker of red blood cell injury is plasma-free haemoglobin (PFHb), which is used for detecting hemolysis. Hemolysis increases non-linearly at lower blood flow rates. When blood flow rates are decreased, there is prolonged blood exposure to artificial surfaces, increasing mechanical stress as well as cellular damage. When the scavenging capacity of haptoglobin is increased, free hemoglobin circulates as dimers and releases free heme. Free haemoglobin scavenges nitric oxide (NO) and converts it to nitrate, impairing its vasodilatory function. This NO depletion results in vasoconstriction as well as increased systemic and pulmonary vascular resistance. It also disrupts platelet and endothelial function, causing platelet aggregation and thrombus formation mediated by von Willebrand factor [5,44].

Free hemoglobin further drives inflammation and promotes a procoagulant state. This cycle worsens hemolysis, depletes clotting factors, and contributes to thrombocytopenia and impaired platelet function, potentially leading to acquired von Willebrand syndrome. Decreasing hemolysis will thus need improved device engineering as well as improved clinical understanding of how operational settings affect blood trauma [5,44].

Thrombosis and bleeding represent parallel and competing risks in ECCO₂R. Anticoagulation is essential for preventing clot formation when blood contacts artificial surfaces, yet it enhances bleeding risk. Shear stress also alters coagulation proteins, compounding this issue. Thrombosis impairs flow in cannulas, pumps, and membrane lungs, increasing mechanical stress and worsening hemolysis. Slow blood flow, particularly in low-shear areas like tubing connectors, promotes thrombin generation and clot initiation. The centrifugal pump head and membrane oxygenator are particularly vulnerable to thrombus formation. Pump thrombosis is usually related to acute and severe hemolysis, whereas membrane thrombosis decreases gas exchange efficiency [45].

Low blood flow rates (1-1.5 L/min) are related to significantly increased hemolysis compared with higher flows around 4 L/min. In low-flow systems, citrate anticoagulation has demonstrated lower hemolysis and decreased platelet loss than heparin. Citrate preserves red blood cell integrity and membrane stability and demonstrated favorable outcomes in animal studies, with minimal thrombus formation even at low flows. Microscopic analysis demonstrated less fibrin and cellular adhesion on membranes with citrate than with heparin [46].

In spite of these advantages, heparin is the most commonly used anticoagulant because of concerns about citrate toxicity. Citrate anticoagulation is reported to be associated with progressive hypocalcemia, even with calcium supplementation, specifically when hemofilter removal of excess citrate is absent. This increases the risk of systemic citrate accumulation and toxicity. Additional studies are required for improving citrate safety profiles in clinical practice [5].

Alternative anticoagulants are also under assessment. Citric acid provides calcium chelation, platelet inhibition, and regional anticoagulation through blood acidification, potentially increasing CO_2_ removal. However, limited hepatic citrate clearance limits achievable blood flows [47]. Other agents like nafamostat mesylate, bivalirudin, and argatroban may be appropriate for patients with elevated bleeding risk or heparin-induced thrombocytopenia. However, cost and pharmacokinetic limitations are still a challenge [5]. Advancements in ECCO₂R will need integrated improvements in device design, anticoagulation strategies, and rigorous clinical validation to establish safer and more effective treatment strategies for critically ill patients [5].

ECCO₂R technology is rapidly evolving, with newer innovations aimed at improving efficiency, safety, and portability. Building on earlier systems like IVOX (intravascular oxygenator), recent designs focus on enhancing gas exchange directly within intravascular catheters. The Hattler catheter improves CO₂ removal through active mixing: a central balloon pulsates within a rigid fiber mat, directing blood flow across membrane fibers and nearly doubling CO₂ exchange rates [27]. Another approach involves rotating fiber bundles in dynamic intravascular lung assist devices, which have demonstrated promising CO₂ clearance, though concerns remain about potential vessel wall injury [8].

Biochemical innovations are also advancing the field. Covalent immobilization of carbonic anhydrase on hollow fiber membranes accelerates bicarbonate conversion to CO₂, significantly enhancing removal efficiency. Other experimental strategies include ultrafiltration of sodium bicarbonate with replacement by sodium hydroxide to improve CO₂ elimination, and loading blood with metabolizable acids such as lactic acid to increase CO₂ tension and facilitate passive diffusion [48].

Additionally, integrated systems combining cardiocirculatory support, membrane oxygenation, and temperature control have been developed for rapid deployment in emergency and transport settings. Wearable ECCO₂R technologies are also progressing, offering continuous CO₂ removal for chronic respiratory conditions and potentially improving long-term mobility and quality of life [8].

Limitations and recommendations

This review has certain limitations. As a narrative review, it does not follow a formal systematic review framework, and therefore, a comprehensive quantitative synthesis or pooled meta-analysis of outcomes was not performed. The included literature comprises a heterogeneous mix of randomized controlled trials, observational studies, and previously published meta-analyses, which may limit direct comparability of findings. In addition, variations in patient populations, device configurations, flow settings, and clinical protocols across studies make it difficult to draw definitive conclusions regarding the overall clinical effectiveness of ECCO₂R. Furthermore, the review was restricted to English-language articles published within the past 15 years, which may have excluded some relevant studies.

Future research should focus on well-designed, large-scale randomized controlled trials and meta-analyses to better define the role of ECCO₂R in acute respiratory failure. Further studies are needed to determine optimal patient selection, timing of initiation, device configurations, and standardized treatment protocols, as well as to evaluate long-term clinical outcomes and safety profiles. Such evidence will be essential in clarifying the precise role of ECCO₂R in modern critical care practice.

## Conclusions

In conclusion, this narrative review highlights that extracorporeal carbon dioxide removal (ECCO₂R) is an emerging but still evolving adjunctive modality in the management of acute respiratory failure (ARF). Current evidence suggests that ECCO₂R provides important physiological benefits, including reduction of hypercapnia, correction of respiratory acidosis, and facilitation of lung-protective ventilation strategies in patients with acute respiratory distress syndrome (ARDS) and acute exacerbations of chronic obstructive pulmonary disease (AECOPD).

However, despite improvements in gas exchange and certain secondary clinical outcomes, a consistent mortality benefit has not yet been demonstrated. In addition, complications such as bleeding and thrombotic events continue to limit its widespread clinical adoption. Variability in study design, patient populations, flow settings, and device technologies further complicates the interpretation and generalization of existing evidence. At present, ECCO₂R should be considered primarily as a selective adjunct or bridging therapy rather than a standard intervention in critical care practice. Future well-designed, large-scale randomized controlled trials are needed to better define appropriate patient selection, optimal timing of initiation, device configurations, and long-term clinical outcomes, thereby clarifying the role of ECCO₂R in modern critical care management.
